# Cognitive dysfunctions in intensive cardiac care unit

**DOI:** 10.4103/0019-5545.64598

**Published:** 2010

**Authors:** Manish Bathla, K. Krishna Murthy, Shalu Chandna

**Affiliations:** Assistant Professor, Department of Psychiatry, M. M. Medical College, Mullana (Ambala), Haryana, India; 1 Professor, Department of Psychiatry, Fr. Muller’s Medical College, Mangalore (Karnataka), India; 2 Reader (Periodontics) M. M. Dental College, Mullana (Ambala), Haryana, India

**Keywords:** Cognitive dysfunctions, heart disease, ICCU

## Abstract

**Background:**

Cognitive impairment is gaining recognition as sequelae of heart failure and the ICCU environment adds to their worsening symptoms.

**Objectives:**

To determine cognitive dysfunctions in patients with heart disease admitted in intensive cardiac care unit (ICCU) and to compare it with patients admitted in general medical wards with heart disease.

**Materials and Methods:**

A total of 30 patients admitted to ICCU with heart disease were taken for the study and compared to patients with heart disease admitted in general medicine wards (except ICCU). The tools used were SMMSE (Standardized Mini Mental State Examination) and BCRS (Brief Cognitive Rating Scale). Statistical tests used were Student ‘t’ test and Chi-Square test.

**Results:**

This study showed cognitive dysfunctions in the domains of orientation, attention and constructional ability as measured by SMMSE and cognitive dysfunction in the domain of concentration as measured by BCRS. Overall cognitive dysfunctions were present in the total score of both SMMSE and BCRS scale, which was statistically very highly significant.

**Conclusion:**

The results showed that the patients in ICCU had cognitive dysfunctions in the domains of orientation, attention, constructional ability and concentration. Overall cognitive dysfunctions were found in the total scores of SMMSE and BCRS, thus signifying a global cognitive deficit.

## INTRODUCTION

Chronic heart failure and cognitive impairment are common problems in the elderly. Both are associated with increased mortality and disability. While these conditions may occur by chance in the same individual, there is increasing evidence that heart failure is independently associated with cognitive impairment.[1]

Cognitive impairment is a common and potentially reversible condition among patients with heart failure, particularly in the elderly. It is described to be associated with an almost five-fold increase in mortality in patients with chronic heart failure.[2]

Both these conditions are associated with increased morbidity, mortality, disability, decrease in quality of life and increased health care costs. Though these conditions may occur together in the same individual, there is increasing evidence that heart failure is independently associated with cognitive impairment. Results show that death occurred in 18% of those who had cognitive impairment with respect to 3% of those with normal cognition. Thus, cognitive impairment is proposed to be an independent prognostic marker in patients with heart failure. Hence simple cognitive screening test should be a part of the routine assessment.[2]

The mechanism of cognitive deficits in cardiac patients is unclear and it may be related to multiple infarcts, acute or chronic hypoxic damage secondary to arrhythmias, cardiac failure, or small vessel disease of the brain.[3]

Congestive cardiac failure is a frequent complication of most diseases of the heart and associated with impairment in several aspects of the quality of life of patients, including mood and cognitive performance. Patients with congestive cardiac failure display deficits in memory and other intellectual abilities, which may be the reason for poor follow-up rates and poor drug compliance.[4]

The mechanism of cognitive deficits in cardiac patients is unclear as it may be related to multiple infarcts/emboli, acute or chronic hypoxic damage secondary to arrhythmias, cardiac failure, hypo perfusion and small vessel disease of brain, but to identify the exact cause in these patients with heart failure is difficult to determine.[5]

Abnormalities of mental functions are common problems in congestive cardiac failure patients, which are more frequent and more serious as the heart failure progresses. Cardiac output and cerebral blood flow are preserved due to compensatory mechanism in mild heart failure but can be severely compromised in advanced heart failure. Drugs used to treat heart failure, especially digitalis, can produce a wide variety of mental aberrations including delirium.[6]

During hospitalization, up to 72% of the patients had mild to severe impairment in one or more cognitive areas, delayed recall being the most common deficit during hospitalization. Six months later, 29% of the patients continued to be impaired and all had deficits in delayed recall.[7] Patients with heart failure experience problems with memory, attention, speed and flexibility of mentation, reaction time and concentration.[8]

The cognitive functions that were most often impaired in patients with chronic heart failure were short-term verbal memory, short term visual spatial memory, differed verbal memory, verbal learning and visual spatial logical ability. The high prevalence of short term verbal memory impairment has important implications in clinical practice since congestive cardiac patients should be actively involved in medical management of their disease. Memory deficits could compromise patient’s adherence to the treatment.[9]

Memory and attention deficits are the most frequently occurring cognitive impairment, followed by slowed motor response times and difficulties in problem solving. Prevalence rates range from 30 to 80%. Most patients have mild impairment, although as many as one fourth may have moderate to severe cognitive impairment. Cerebral infarction and cerebral hypo perfusion either alone or in combination might be the underlying pathophysiology.[1]

Micro embolization and cerebral hypo perfusion were implicated for neurocognitive dysfunction in chronic heart failure. Other causes of cognitive decline may include prior cardiac surgery, chronic hypertension; sleep disordered breathing, hyper homocysteinemia and dementia of ageing. The discovery of neurocognitive defects in heart failure must prompt a well constructed diagnostic evaluation to search for the underlying cause since this process might be at least partially reversible in many cases.[10]

Hemodynamic alterations due to heart failure and cognitive deteriorations are very frequently associated in aging, increasing morbidity and mortality risk. Psychosocial variables such as illiteracy, depression and particularly cognitive deterioration determine a significant increase of the risk to develop heart failure. Attention should be paid to encourage mild physical activity to provide emotional support to patients and also to assess their general cognitive abilities. Hence multidimensional approach is necessary to better characterize and treat elderly patients in particular those with congestive cardiac patient.[11]

Low-output states such as systemic hypotension[12] and low ejection fraction[8] also have been implicated as causes of cognitive impairment in patients with heart failure.

Abnormal prevalence of cognitive dysfunction has been reported in middle aged patients with end stage heart failure. Increasing age and lower indices of left ventricular function were associated with worsening cognitive performance. However, the linkage between ageing, left ventricular systolic function, and cognitive performance remains elusive.[12] Cognitive impairment may be more common in patients with heart failure than in the general population.[13] Other investigators have reported that 23 to 53% of patients with heart failure have evidence suggestive of cognitive impairment.[12] The risk of cognitive impairment in patients with heart failure was 1.96 times the risk in the general population, 65 years or older.[14]

Arterial hypotension has been associated with increased risk of dementia in some large prospective studies; and cognitive impairment is common among elderly with left ventricular function. As early treatment of cardiac low-output states can reverse cognitive dysfunction, the routine management of heart failure should include systematic assessment of cognitive performance.[13]

In patients with heart failure, cognitive impairment may be intermittent or subtle in the early stages and thus not easily recognized. If so, routine screening is essential to ensure that cognitive impairment is detected and addressed as quickly as possible. In order to do so, practical, time-efficient, and sensitive measures are needed. Cognitive screening tools should be tested against a reference standard, such as a clinical neuropsychological battery, so that the sensitivity and specificity of simple measures for detecting cognitive impairment in patients with heart failure could be identified.[15]

Systematic neuropsychological testing of older patients with heart failure for early diagnosis of cognitive impairment might identify those who may most benefit from prompt echocardiographic evaluation and aggressive treatment of left ventricular dysfunction. Such a multidisciplinary approach to older patients with heart failure may play a key part in reducing the burden of so called "circulatory dementia" in advanced age, as the prevalence and incidence of heart failure are rapidly increasing, and substantial decreases in cognitive function have been associated in general populations with diagnosis of cardiovascular diseases.[16]

### Objectives

To determine cognitive dysfunctions in patients with heart disease admitted in ICCU and compare with patients admitted in general medical wards with heart disease.

### Methodology

Subjects for the study were selected from the inpatient facility of Department of General Medicine, Father Muller’s Medical College, Mangalore (Karnataka).

### Method of Collection

Thirty subjects diagnosed as having cardiac disease, admitted in ICCU, were chosen randomly. Initial contact was made in ICCU after three days of admission. An informed consent was obtained from those who were willing to participate in the study. Investigations; like Liver Function Tests (LFT) to rule out any liver disease, Renal Function Tests (RFT) to rule out any renal disease, Random Blood Sugar (RBS) for diabetic status, Serum Electrolytes, ECG and Cardiac Enzymes - CPK, CKMB; were done to rule out systemic disorders which may be causative of cognitive dysfunctions.

### Inclusion Criteria

1. Patient admitted in ICCU with heart disease
2. Patient admitted in general medical wards with heart disease
3. Age: 18-64 years
4. Patients who stayed for at least three days

### Exclusion Criteria

1. Patients < 18yrs and >64 years
2. History of substance use within one week prior to admission except tobacco and social use of alcohol
3. Any psychiatric consultation in last one month
4. Patient with known history of any chronic organic mental illness
5. Patients with multiple chronic diseases causing cognitive impairment like neuro-degenerative disease, thyroid and adrenal disorders, renal disorders, cancers and stroke
6. Patients who were critically ill and who cannot participate in the study like patients on respiratory or ventilatory support

After screening, the patients who met the inclusion criteria were taken into the study for detailed assessment.

### Tools and Instruments

1. Semi – Structured Proforma (specially designed for this study)
2. Standardized Mini-Mental State Examination (SMMSE)[17]
3. Brief Cognitive Rating Scale (BCRS)[18]

**Type of Study:** This is a cross sectional study.

## RESULTS

Results of the study observed are as shown in Table - 1, 2, 3, 4, 5.

**Table 1:** Demographic variables

Demographic variables		Controls	Cases
Sex	Male	21	21
	Female	09	09
Marital status	Married	30	30
	Single	00	00
Religion	Hindu	10	13
	Muslim	07	12
	Christian	13	05
Domicile	Urban	09	06
	Rural	21	24
Education	Primary	22	22
	Secondary	06	06
	Higher secondary	01	01
	Graduate	01	01
Income data	<2500	18	18
	2500-4999	09	09
	5000-7499	02	02
	>7500	01	01
Occupation status	Professional	00	01
	Manager	01	00
	Clerk	02	00
	Business	04	03
	Farmer/coolie	08	08
	Industry worker	02	00
	Service	00	01
	Retired	02	03
	Housewife	05	08
	Non agricultural	06	06
	workers		

**Table 2:** Medical diagnosis

	Group	
	
	Controls	Cases
Accelerated hypertension	8	0
	26.7%	0%
Congestive cardiac Failure	1	3
	3.3%	10.0%
Myocardial infarction	14	15
	46.7%	50.0%
Severe left ventricular failure	0	2
	0%	6.7%
Stable angina	7	0
	23.3%	0%
Unstable angina	0	10
	0%	33%

**Table 3:** SMMSE score

	Group	Mean	Std. deviation	T
Orientation	Controls	9.06	1.01	3.4050
	Cases	7.70	1.96	*P*=.001 vhs
Registration	Controls	2.80	0.407	1.4330
	Cases	2.63	0.490	*P*=.157 ns
Attention	Controls	3.97	1.36	3.6230
	Cases	2.83	1.05	*P*=.001 vhs
Recall	Controls	2.63	0.49	1.683
	Cases	2.33	0.84	*P*=.098 ns
Language	Controls	7.70	0.65	1.6110
	Cases	7.36	0.92	*P*=.113 ns
Construction	Controls	0.56	0.50	2.1280
	Cases	0.30	0.46	*P*=.038 sig
Total score	Controls	26.73	2.71	3.6000
	Cases	23.16	4.69	*P*=.001 vhs

**Table 4:** BCRS score

	Score	Group	
	
		Controls	Cases
Concentration	1.00	23	7
		76.7%	23.3%
	2.00	4	4
		13.3%	13.3%
	3.00	3	6
		10.0%	20.0%
	4.00	0	5
		0.0%	16.7%
	5.00	0	4
		0.0%	13.3%
X2 = 22.533	6.00	0	4
*P* = . 001 Vhs		0.0%	13.3%
Recent memory	1.00	30	26
	2.00	0	3
		0.0%	10.0%
X2=4.286	3.00	0	1
*P* = .117 Ns		0.0%	3.3%
Past memory	1.00	30	30
*P* = 1 ns		100.0%	100.0%
Orientation	1.00	27	28
		90.0%	93.3%
*P* = 1 ns	2.00	3	2
		10.0%	6.7%
Functioning and self care	1.00	30	28
		100.0%	93.3%
*P* = .492 ns	2.00	0	2
		0.0%	6.7%

**Table 5:** BCRS total score

Group	Mean	Std. deviation	Z
Controls	1.08	0.18	4.3610
Cases	1.50	0.44	*P* =.001 vhs

## DISCUSSION

Table 1 shows the demographic variables.

A significant number (73.3%) of patients in this study were assessed on the third day of admission following which they were noticed to be having cognitive dysfunctions.

The cognitive dysfunctions of the intensive cardiac care unit (ICCU) patients in the present study were comparable to those found at the time of hospitalization in a previous study, conducted by Sauve *et al*.[7]However, our study was not comparable to other studies which had found cognitive dysfunctions at various time gap of six weeks;[19] three months[20] as ours is a cross sectional study and no follow-up data is available.

In this study, majority of the patients were diagnosed as having myocardial infarction (50%), unstable angina (33.3%) and congestive cardiac failure (10%). No other previous studies have mentioned the commonest medical diagnosis in their sample[Table 2].

On SMMSE cognitive domains like orientation and attention were statistically very highly significant and the domain of construction was statistically significant [Table 3]. The domain of orientation was comparable with the results in the study done by Barclay *et al*.[3]and Sauve *et al*.[7] The domain of attention was comparable with the results in previous study done by Sauve *et al*,[7]Almeida *et al*.[19] and Bennet *et al*.[1] but was not comparable with another study by Dijkstra *et al*.[21] which showed that attention and speed related cognitive functions were not affected. The domain for constructional ability was comparable to the previous study done by Callegari *et al*.[9]

The SMMSE scale did not show any statistical significance in the domains of registration, recall and language [Table 3]. This was not comparable to the results shown in the previous studies on the domains of recall[1,3,7] and registration,[9] which may be because of the different test scales used in these studies. No other previous study have mentioned about language functions.

Results of the BCRS scale showed a statistically very highly significant deficit in the domain of concentration [Table 4]. This was comparable to a previous study by Sauve *et al*.[7]

Other domains assessed using BCRS scale such as recent memory, past memory, orientation and functioning and self-care were statistically not significant [Table 4]. This was comparable to a previous study by Grubb *et al*,[22] which showed no memory impairment. This was not comparable with previous studies for the domains of recent memory and past memory[1,3,7,9] and also to a study by Dijkstra *et al*[21] which showed that depressed myocardial infarction patients performed better with respect to memory. The domain of orientation was not comparable with previous studies done by Barclay *et al*[3] and Sauve *et al*[7] which found that orientation was impaired. No other previous studies have mentioned regarding the domain of functioning and self-care.

Finally, in the SMMSE scale and BCRS scale the overall total scores in cases showed results, which were statistically very highly significant [Tables 4 and 5]. This was similar to the results noticed in many of the previous studies of overall cognitive decline.[15,19,23]

## CONCLUSION

- The results of socio-demographic variables showed that the mean age was 51.53 years, majority being males, married, residing in rural areas, educated up to primary school and earning less than Rs. 2,500/- per month.
- Occupational background showed that there were 26.7% farmers / coolies, 26.7% housewives and 20% non-agricultural laborers.
- SMMSE score showed deficits in the domain of orientation, attention and constructional ability.
- BCRS score showed deficits in the domain of concentration.
- Overall cognitive decline was observed on both SMMSE and BCRS scores.
- Signifies the presence of global cognitive deficit.

### Limitations

- This study was limited by the small sample size
- Subgroup analysis was not done
- Other etiological factors of cognitive impairment like – age, severity of illness, medications, nutritional deficiencies, depression, infection and fluid shifts were not considered.

### Clinical Implications

This was a cross sectional study with a small sample size, but the study reveals that patients with heart failure in ICCU had more cognitive impairments than the patients in general medical wards. This is supported by the data from earlier studies. These cognitive states may be reversible if detected early, as is supported by the data.

### Suggestions

Future research needs to focus on:

1. determining the types, frequency, and severity of impairments in cognitive functioning among a sample of heart failure patients,
2. explicating the pathological mechanisms and the clinical factors that underlie the development of cognitive deficits,
3. identifying the ways cognitive impairment influences quality of life, and
4. follow-up studies; studies assessing cognitive dysfunction at six months and one year follow-up may be helpful as they may be correlated with the severity of heart disease, presence of depression and permit the clinician to take decision in the treatment issues.
